# Identifying Oneself with the Face of Someone Else Impairs the Egocentered Visuo-spatial Mechanisms: A New Double Mirror Paradigm to Study Self–other Distinction and Interaction

**DOI:** 10.3389/fpsyg.2016.01283

**Published:** 2016-08-25

**Authors:** Bérangère Thirioux, Moritz Wehrmann, Nicolas Langbour, Nematollah Jaafari, Alain Berthoz

**Affiliations:** ^1^Laboratoire de Physiologie de la Perception et de l’Action UMR 7152 CNRS, Collège de FranceParis, France; ^2^Unité de Recherche Clinique Intersectorielle en Psychiatrie à vocation régionale Pierre Deniker, Centre Hospitalier Henri LaboritPoitiers, France; ^3^Bauhaus-Universität WeimarWeimar, Germany; ^4^Université de Poitiers – INSERM CIC-P 1402 du CHU de Poitiers – INSERM U 1084, Experimental and Clinical Neuroscience Laboratory – Groupement de Recherche CNRS 3557Poitiers, France

**Keywords:** self-face identification, bodily self-consciousness, visuo-spatial mechanisms, self–other distinction

## Abstract

Looking at our face in a mirror is one of the strongest phenomenological experiences of the Self in which we need to identify the face as reflected in the mirror as belonging to us. Recent behavioral and neuroimaging studies reported that self-face identification not only relies upon visual-mnemonic representation of one’s own face but also upon continuous updating and integration of visuo-tactile signals. Therefore, bodily self-consciousness plays a major role in self-face identification, with respect to interplay between unisensory and multisensory processing. However, if previous studies demonstrated that the integration of multisensory body-related signals contributes to the visual processing of one’s own face, there is so far no data regarding how self-face identification, inversely, contributes to bodily self-consciousness. In the present study, we tested whether self–other face identification impacts either the egocentered or heterocentered visuo-spatial mechanisms that are core processes of bodily self-consciousness and sustain self–other distinction. For that, we developed a new paradigm, named “Double Mirror.” This paradigm, consisting of a semi-transparent double mirror and computer-controlled Light Emitting Diodes, elicits self–other face merging illusory effect in ecologically more valid conditions, i.e., when participants are physically facing each other and interacting. Self-face identification was manipulated by exposing pairs of participants to an Interpersonal Visual Stimulation in which the reflection of their faces merged in the mirror. Participants simultaneously performed visuo-spatial and mental own-body transformation tasks centered on their own face (egocentered) or the face of their partner (heterocentered) in the pre- and post-stimulation phase. We show that self–other face identification altered the egocentered visuo-spatial mechanisms. Heterocentered coding was preserved. Our data suggest that changes in self-face identification induced a bottom-up conflict between the current visual representation and the stored mnemonic representation of one’s own face which, in turn, top-down impacted bodily self-consciousness.

## Introduction

The distinction between self and others is fundamental for social interactions. In fact, under non-pathological conditions, a balanced relationship between two individuals equally preserves the processing of the Self and that of the Other. The Self is a wide concept that includes, non-exhaustively, multidimensional episodic, semantic but also physical personal representations and associated perceptual-cognitive processes (Duval et al., 2007). In this study, we focused on the physical Self and, especially, on two of its fundamental phenomenological features. That is, self-face identification and bodily self-consciousness. Self-face identification refers to one’s ability to identify with the image of one’s own face as reflected in the mirror (Rochat, 2003). Bodily self-consciousness refers to one’s normal experience of the spatial unity between the Self and the body (Blanke, 2012).

Self–other distinction already operates on a lower-order visual level in self-face identification. This is reflected in the so-called self-face prioritization (SFP) effect in which recognizing one’s own face, compared to unfamiliar (Tong and Nakayama, 1999) and familiar faces (Sugiura et al., 2005, 2006, 2008; Ma and Han, 2010; Sui et al., 2015), always triggers faster response speed and better performances. Self–other distinction also operates on the visuo-spatial level in bodily self-consciousness. That is, visuo-spatial mechanisms, respectively, centered on one’s own-body (egocentered) and the other’s body (heterocentered) (Degos et al., 1997; Cleret de Langavant et al., 2011) enable dissociating between self and others in space (Thirioux et al., 2010). These visuo-spatial processes are involved in perspective-change and lie at the basis of higher-order self–other distinction such as in empathy (Ruby and Decety, 2001, 2003; Frith and Frith, 2003; Decety and Jackson, 2004; Thirioux et al., 2014a).

Until only recently, the modular approach has prevailed in experimental research on physical self. It was assumed that self-face identification and bodily self-consciousness are sustained by different perceptual-cognitive mechanisms. That is, self-face identification would be based upon unisensory and mnemonic processing whereas bodily self-consciousness upon multisensory processing and continual updating of body-related information. However, behavioral and neuroimaging studies have newly reported that self-face identification continually updates body-related information and, specifically, integrates visual and tactile signals (Tsakiris, 2008; Sforza et al., 2009; Paladino et al., 2010; Tajadura-Jiménez et al., 2012; Apps et al., 2015). However, although visuo-spatial mechanisms are a core process of bodily self-consciousness and require the integration of visual, proprioceptive and vestibular signals, there is so far no data regarding the relation between self-face identification and visuo-spatial processing.

### Multisensory Integration and Visuo-spatial Mechanisms in Bodily Self-consciousness

Self-identification with the body (the experience of owning a body), self-location (the experience of where I am in space) and first-person perspective (the experience from which I perceive the world) are three main components of bodily self-consciousness (Blanke and Metzinger, 2009). Self-identification integrates somatosensory and visual signals whilst self-location and first-person perspective additionally integrates vestibular information (Blanke, 2012). The integration of retinal, proprioceptive, auditory and vestibular inputs in association with motor outputs and body movements generate information related to the body position in space and an egocentered referencing system (Berthoz et al., 1975; Berthoz, 1988, 1997; Ventre-Dominey et al., 2003; Giummarra et al., 2008). Concordant with the phenomenological features of bodily self-consciousness, specific activations at the right temporo-parietal junction (TPJ) in the vestibular system (Kahane et al., 2003) have been reported to support self-location and first-person perspective (Blanke et al., 2004; Ionta et al., 2011).

The continual updating and integration of body-related signals further enable encoding the other’s body position in space and distinguishing between self and others. In fact, experiencing the visuo-spatial perspective of another individual requires imagining one’s own-body into the other’s body position (Vogeley and Fink, 2003). This heterocentered coding necessitates geometrical transformations including translations and rotations of one’s viewpoint (Deroualle and Lopez, 2014). It also requires multisensory integration based on mental simulation. It means that encoding the visuo-spatial perspective of someone else relies upon the mental simulation of visual, proprioceptive (sensory perception of the position of one’s body parts) and vestibular (self-motion) self-related processes (Deroualle and Lopez, 2014). It has been recently shown that self–other distinction involves parallel egocentered and heterocentered visuo-spatial coding, respectively, in the right and left TPJ (Thirioux et al., 2010, 2014a; see also Berthoz, 2004). This co-processing would operate in association with computational mechanisms that decouple between first- and second-person signals in the right dorsolateral prefrontal cortex (dlPFC; Thirioux et al., 2014a; see also Aichhorn et al., 2006; Mitchell et al., 2006; McCleery et al., 2011).

Interestingly, data from neurological patients with lesions at the TPJ who experienced out-of-body experience (Irwin, 1985; Devinsky et al., 1989; Brugger et al., 1997; Blanke et al., 2004) or heautoscopy (Menninger-Lerchenthal, 1935; Hécaen and Ajuriaguerra, 1952; Brugger, 2002), i.e., pathological embodiment and self-location, evidenced how the multisensory integration of body-related information fundamentally contribute to body ownership, self-location and first-person perspective. In healthy subjects, behavioral studies report that multisensory conflict between visual, proprioceptive, vestibular, and tactile information alters bodily self-consciousness and disturbs self–other distinction (Ehrsson, 2007; Lenggenhager et al., 2007; Ionta et al., 2011). Stroking the back of participants synchronously with the back of a virtual body presented through a head-mounted display elicited self-identification with the virtual body (Ehrsson, 2007; Petkova and Ehrsson, 2008; Olivé and Berthoz, 2012) and modifications in self-location. That is, participants had the impression to be located at a position in space that was outside their physical position and toward that of the virtual body (Lenggenhager et al., 2007; Aspell et al., 2009). Moreover, synchronous visuo-tactile stimulation, when a virtual body was seen from a visuo-spatial perspective matching the participants’ egocentered perspective, generated stronger self-identification with the virtual body, in comparison to the second-person perspective (heterocentered; Slater et al., 2010; Petkova et al., 2011).

Taken together, these data firstly indicate that the integration of visual, somatosensory, proprioceptive, and vestibular signals is necessary to encode one’s own-body position in space and egocentered visuo-spatial perspective. Secondly, these data point out that this multisensory integration is also necessary to mentally simulate the visuo-spatial perspective of another individual and distinguish between self and others.

### Multisensory Integration of Bodily Related Signals in Self-face Identification

Behavioral research has until now mostly focused on the contribution of visual processing and mnemonic representations of one’s own face to self-face identification (Tong and Nakayama, 1999; Brédart, 2003; Brady et al., 2004, 2005). In addition to higher-order semantic processes enabling to access an amodal representation of the self, these mechanisms validate the knowledge that the individual as seen in the mirror is the self (Morin, 2007; Devue and Brédart, 2011; Sugiura et al., 2012, 2014). However, self-face identification has been recently shown to also integrate visual and tactile information, similarly to bodily self-consciousness (Tsakiris, 2008; Sforza et al., 2009; Paladino et al., 2010; Apps et al., 2015). This has been demonstrated by causing illusory self-identification with the face of another individual while using interpersonal multisensory stimulation (IMS) in healthy volunteers (Tajadura-Jiménez et al., 2012). Especially, synchronous visuo-tactile stimulation between one’s own face and a morphed face consisting of 50% of the participant’s face and 50% of the face of someone else was found to alter self-face recognition judgments. That is, after IMS, participants assessed images of morphed faces with more other- than self-face physical features as containing higher percentage of self- than other-face features (Tsakiris, 2008; Sforza et al., 2009). Therefore, participants incorporated the features of the other’s face into the representation of their own face (Maister et al., 2013). This “enfacement experience” – i.e., the experience to see and feel the other’s face as one own face (Tsakiris, 2008) – shows that IMS, affecting multisensory integration processing, alters self–other distinction.

Data from low frequency repetitive transcranial magnetic stimulation (rTMS) studies corroborated the role of body-related signals integration in self-face identification. Stimulation over the right but not left TPJ has been reported to impair visual self–other discrimination (Uddin et al., 2006). In this case, morphed images with 60% of other-face physical features were evaluated as containing more “self” than “other.” Heinisch et al. (2011), using a video-based morphing technique, further specified that rTMS over the right TPJ biases self–other distinction toward less conservative self-recognition performance. Other-face recognition was preserved.

In line with these results, the perception of current but not past images of one’s own face has been found to activate the inferior occipital gyrus (IOG) in association with the inferior temporal gyrus (ITG) and TPJ. These co-activations indicate that the visual representation of one’s facial appearance is continuously updated (Apps et al., 2012). Therefore, multisensory integration in facial self-consciousness would underpin both self-identification and self-updating (Apps et al., 2012). Moreover, in an fMRI study employing IMS and visual self–other discrimination tasks, the intensity of the enfacement experience was associated with an IOG activation that positively co-varied with a decrease of the BOLD signal in the TPJ (Apps et al., 2015). It means that the modulation of the self–other distinction in the TPJ affected the visual representation of one’s own face in the IOG and, consequently, self-identification.

Collectively, these data demonstrate that the interplay between unisensory and multisensory processing and updating lies at the basis of both self-face identification and distinction between one’s own and other’s face (Apps et al., 2015).

### Limitations and Hypotheses

Overall, these studies provide important insights into how self-face identification relates to multisensory integration of body-related signals. However, these data need to be further expanded for four main reasons.

Firstly, if egocentered and heterocentered visuo-spatial mechanisms continuously update bodily self-consciousness, there is so far no data regarding how self-face identification relates to the integration of visual, proprioceptive, and vestibular signals. In fact, previous studies have mainly focused on the relation between self-face identification and integration of visuo-tactile information.

Secondly, prior studies investigating the interplay between unisensory and multisensory processing in self-face identification used experimental paradigms that are based upon induced multisensory conflicts. Especially, these studies addressed the question whether visuo-tactile conflicts affect the visual representations of one’s own face (multisensory → unisensory). However, if the hypothesis that there is an interplay – i.e., a bidirectional interaction – between multisensory and unisensory processing in self-face identification is suitable, then experimental paradigms should also test whether an induced unisensory conflict affects the multisensory processing (unisensory → multisensory). To the best of our knowledge, there is until now no data regarding how self-identification with the other’s face in a mirror (i.e., induced visual conflict) impacts the visuo-spatial referencing systems.

Thirdly, if a dynamic switch between the egocentered and heterocentered perspective preserves self–other distinction on a visuo-spatial level, it is still unknown whether self-identification with the other’s face impacts either the egocentered or heterocentered visuo-spatial mechanisms.

Fourthly, previous studies most often investigated the relation between self-face identification and multisensory integration when an individual was presented with pre-recorded images or videos of his/her own face that was morphed with the face of someone else. That is, these studies did not use real-time self–other face morphing or merging illusion when two individuals are facing each other and interacting. Although of high technology quality, these setups render therefore the experimental conditions less ecologically valid.

**FIGURE 1:**
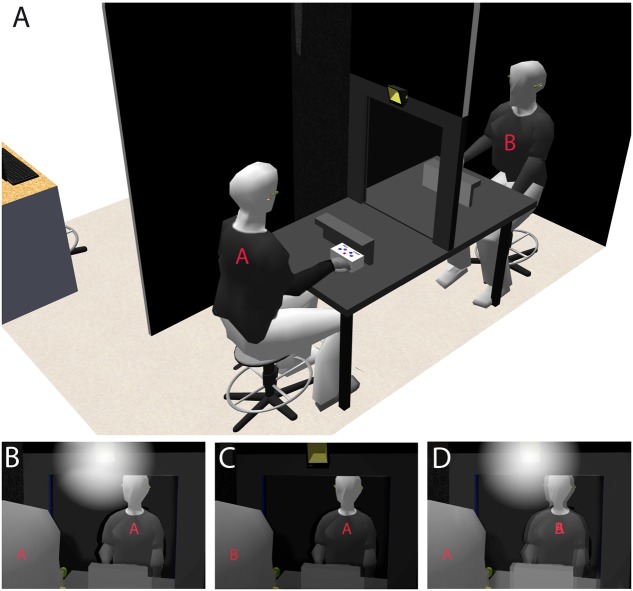
**The Double Mirror system.** **(A)**, Two individuals, *A* and *B*, are facing either side of the semi-transparent double mirror. A set of five white LEDs is fixed in the middle of the upper edge of each mirror’s side. **(B)** If the LEDs set on *A*’s side is on whereas that on *B*’s side is off, *A* can see his/her own face reflected in the mirror without seeing *B*’s face through the mirror. **(C)** In contrast, while using the same lighting mode, *B* can see *A*’s face through the mirror without seeing his/her own face reflected in the mirror (and *vice-versa* if the LEDs set on *A*’s and *B*’s side are, respectively, off and on). **(D)** If both LEDs sets are simultaneously on and alternatively flickering at a frequency of 8 Hz, the reflections of *A*’s and *B*’s faces are merging in the mirror.

Here, we aimed to investigate whether identifying oneself with the face of another individual impacts (i.e., in terms of impairment and/or improvement) the egocentered or heterocentered visuo-spatial mechanisms that sustain self–other distinction. For that, we developed a new paradigm, named “Double Mirror,” that enables testing self-consciousness by exposing two individuals to Interpersonal Visual Stimulation (IVS) in ecologically more valid conditions, i.e., when individuals are physically facing each other and interacting. We used an innovative technology that is based upon a semi-transparent double mirror and a set of five computer-controlled Light Emitting Diodes (LEDs) that is fixed in the middle of the upper edge of each mirror’s side. If two individuals, *A* and *B*, are facing either side of the mirror and only the LED set on *A*’s side is on, *A* can see his/her own face reflected in the mirror but without seeing *B*’s face through the mirror (Self-condition). Simultaneously, *B* can see *A*’s face through the mirror but without seeing his/her own face reflected in the mirror (Other-condition; and *vice-versa* if only the LED set on *B*’s side is on). If the two LED set are alternatively flickering at a frequency of about 8 Hz, the reflections of the two individuals’ faces are merging into the mirror, inducing self-identification with the other’s face and the striking phenomenological experience of making one with the other (**Figure 1**).

The present experiment consisted of three phases: pre-stimulation test (phase 1), IVS (phase 2) and post-stimulation test (phase 3). We tested pairs of healthy volunteers who were facing each other and interacting. The two participants simultaneously performed visuo-spatial and mental own-body transformation tasks either on themselves (Self-condition) or their facing partner (Other-condition) before and after exposure to IVS. Tasks were performed with respect to green flashes that were emitted by markers placed symmetrically on the participants’ left and right temples and that appeared randomly. In the Self-condition, participants were instructed to assess whether the green flash on their face as reflected in the mirror was physically appearing on the left or right side of their own face (egocentered visuo-spatial mechanisms). In the Other-condition, participants assessed whether the green flash was physically appearing on the left or right side of their partner’s face seen through the mirror (heterocentered visuo-spatial mechanisms). In the IVS-phase, participants judged whether the green flash on the self–other morphed face as reflected in the mirror was physically appearing on the left or right side of their own face or the face of their partner.

There were four possible patterns of errors regarding self- vs. other-face features attribution in the IVS-phase. That is, participants could either over-attribute the features of their own face to the face of their partner (Self → Other) or over-attribute the features of their partner’s face to their own face (Other → Self). These further distinguish into over-attribution in reflection symmetry (Thirioux et al., 2009; i.e., in mirror reversal; Brugger, 2002) and rotation symmetry (Thirioux et al., 2009; i.e., with preservation of the lateral asymmetry; Brugger, 2002). We aimed to analyze the different errors patterns in the IVS-phase because (1) we hypothesized that a dominant pattern of attribution error in reflection symmetry indicates that the self–other face merging illusion is effective. That is, this pattern would reflect that individuals *identified* themselves with the reflection of the other’s face. In fact, this illusory felt correspondence between the kinesthesic representation of one’s own face and the reflection of the other’s face in the mirror would resemble the normal matching between the kinesthesic and physical representation of one’s own-face and the image of one’s own face in a mirror. (2) We further hypothesized that these different patterns of attribution errors would have a different impact on the egocentered and heterocentered mechanisms in the post-stimulation test. We expected that a prevailing attribution pattern in reflection symmetry for Self-trials (Self → Other) and Other-trials (Other → Self) would, respectively, impair and improve the egocentered coding in the post-stimulation test, in comparison to the pre-stimulation test. In contrast, we expected that a prevailing attribution pattern in rotation symmetry for Self-trials (Self → Other) and Other-trials (Other → Self) would, respectively, improve and impair the heterocentered coding in the post-stimulation test.

Our data show that self–other face identification impaired the egocentered visuo-spatial mechanisms. In contrast, heterocentered visuo-spatial mechanisms were preserved. This suggests that changes in self-face identification induced a bottom-up mismatching between the current visual representation of one’s own face and the stored mnemonic representation of the self-face which, in turn, top-down impacted bodily self-consciousness.

## Materials and Methods

### Participants

Nine pairs of healthy volunteers took part in this experiment. In order to generate a more robust self–other face merging illusory effect, we tested same-sexed pairs of participants. We excluded the data from four participants because of bad recording conditions during the IVS-phase (for more details, see Post-stimulation Test). For the final analysis, we included, thus, the data from the remaining fourteen participants (six women, eight men; aged 19–32 years; mean ± SD 22.1 ± 3). All participants were right-handed according to the Edinburgh handedness inventory (Oldfield, 1971). All participants had normal vision, i.e., without correction. Thus, individuals with eyeglasses for vision correction or contact lens were systematically excluded from participation. None reported history of neurological or psychiatric disorders.

### Ethics Statement

All volunteers were naïve to the purpose of the experiment, gave written informed consent and were given with monetary compensation of 10 € after the experiment. The study protocol has been approved by the local ethics research committee and performed in accordance with the ethical standards laid down in the Declaration of Helsinki.

### Experimental Setup

#### Stimulus and Apparatus

##### The “Double Mirror” paradigm

We used the Alter Ego System that was designed and programmed by Moritz Wehrmann^©^. We here introduce the term “Double Mirror” to refer to the Alter Ego System. This consists of a semi-transparent double mirror (70 cm × 50 cm × 0.4 cm; height × width × depth) and two sets with five white LEDs each. A LED set is fixed in the middle of the upper edge of each mirror’s side (**Figure 1**). These LED sets can [1] emit continuous light either separately (i.e., only one of the two sets is on) or simultaneously (i.e., both sets are on) or [2] flicker alternatively at a given frequency.

These different using modes of lighting enable generating, when two individuals – *A* and *B* – are facing either side of the mirror, different Self-face and Other-face perceptual conditions (**Figure 1A**). These lighting modes determined our three experimental conditions and associated tasks (see Tasks). [1] If the LED set on *A*’s side is on (*A*’s face is illuminated) whereas that on *B*’s side is off (*B*’s face is not illuminated), *A* can see his/her own face reflected in the mirror (as in a usual mirror) but without seeing *B*’s face through the mirror. We here refer to this perceptual condition as **Self-condition** (**Figure 1B**). [2] Using this same lighting mode, B can see *A*’s face through the mirror (as through a window) but without seeing his/her own face reflected in the mirror. We refer to this perceptual condition as **Other-condition** (**Figure 1C**). And *vice-versa*, if the sets on *A*’s and *B*’s sides are, respectively, off and on.

[3] If the two LED sets are simultaneously on (both *A*’s and *B*’s faces are illuminated), the reflections of *A*’s and *B*’s faces are merging in the mirror. In this case, *A* can see his/her own face being merged with *B*’s face, and reciprocally for *B* (**Figure 1D**). This illusory effect of self-identification with the other’s face is further reinforced if the LED sets are alternatively flickering. We refer to this condition as **IVS-condition**.

**FIGURE 2:**
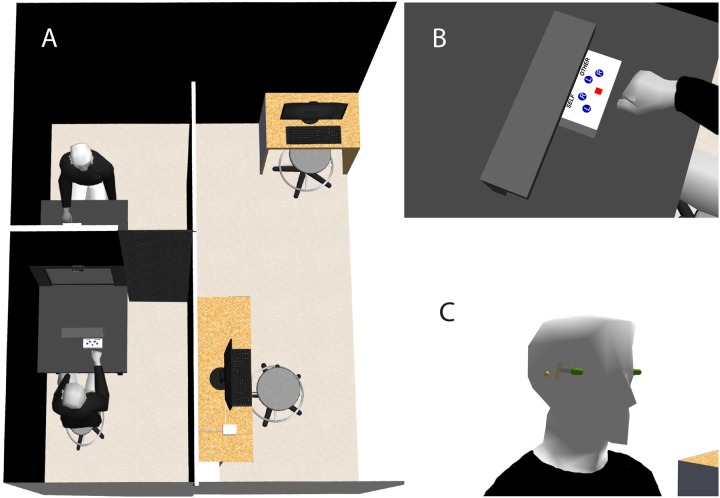
**Experimental setup and procedures.** **(A)** We set up an entirely darkened enclosed area inside the testing room. The mirror was fixed transversally in the middle of a table and built in black curtain parts, splitting the enclosed area into two smaller areas. Each participant was seated on a stool at each end of the table in one dedicated area and facing his/her partner. **(B)** Participants used a serial response box with four mute response buttons that was placed in front of them and behind a black wood cover. **(C)** We placed two markers light, emitting green light, symmetrically on the participants’ left and right temples. Participants performed visuo-spatial transformation tasks with respect to green flashes that were emitted by the markers. Participants further wore B-Brand polyurethane ear plugs that prevented them from synchronizing their response with that of their partner.

Both sampling switch between the two LED sets and the flicker frequencies range (1–20 Hz) were controlled by a PC using E-Prime software (Psychological Software; **Figure 2A**).

##### Handling procedures

The experiment took place in an entirely darkened enclosed area that was set up inside the testing room. The mirror was fixed transversally in the middle of a black plywood table (130 cm × 80 cm). Furthermore, the mirror was built in black curtain parts that extended vertically until the ceiling and laterally until the adjacent walls, splitting the enclosed areas into two smaller areas (**Figure 2A**). Each participant was seated on a stool at each end of the table in one dedicated area and facing his/her partner (distance between the participants’ faces: 130 cm; **Figures 1A** and **2A**). Participants faced one side of the mirror and a serial response box with four response buttons (**Figure 2B**). Each response box was fixed on the table behind a black wood cover so that participants were not able to see through the mirror the fingers’ movements of their facing partner and which response button was pressed (**Figures 1A** and **2A**). To prevent Self- and Other-recognition biases, participants wore black long sleeve shirts and had removed all personal belonging (such as jewelry, scarf, watch, etc.). Participants with long hair were instructed to tie back their hair with an elastic band. We manually adjusted the stool’s height of each participant so that the reflections of the participants’ eyes were at the same level and merged in the mirror. This was done while the two LED sets were simultaneously on (without flickering). Using hypo-allergenic and transparent Band-Aid^TM^, we placed two markers light, emitting green light, symmetrically on the participants’ left and right temples (**Figure 2C**). These marker lights were controlled with a PC using E-Prime software. Because we used a semi-transparent double mirror, the brightness of the markers when reflected in the mirror in the Self-condition and when seen through the mirror in the Other-condition was equalized. This prevented from self- vs. other- recognition biases in the IVS-condition (see Phase 2: Interpersonal visual stimulation). Then, we made sure that participants could not see in the peripheral vision the green flash emitted by the markers on their temples. For that, participants were instructed to stare at a red point embedded into the mirror (viewing distance: 65 cm) while the markers on their left and right temples were emitting green flashes randomly (duration: 500 ms; mean ISI: 2500 ms; 10 trials (5 [repetitions] × 2 [markers])). Participants were asked to verbally indicate whether and when they have seen in the peripheral vision a green flash emitted by the left or right marker. Markers were considered as being correctly positioned if participants reported to have not seen a green flash during any trial.

Based on previous work (Blanke et al., 2005; Arzy et al., 2006; Mohr et al., 2006; Thakkar et al., 2009; Thirioux et al., 2009, 2010, 2014b), we expected faster reaction times (RTs) when participants performed the visuo-spatial task on themselves than on their facing partner. Consequently, to avoid a response synchronization bias due to that participants performed the tasks simultaneously (see Tasks), we used mute response buttons and participants wore B-Brand polyurethane ear plugs (**Figure 2C**). This prevented participants from hearing when the response button was pressed by their facing partner and, thus, from synchronizing their responses. Then, we further checked that the clicking sound was not audible. For that, we instructed participants to keep their eyes closed and to verbally indicate if they have heard a clicking sound while their partner was pressing one of the four response buttons on the response box. They were five repetitions per response button (5 × 4 trials). The button-press was considered as not being audible if participants reported to have heard no sound during any trial.

#### Tasks

To test whether self–other face identification impacts either the egocentered or heterocentered visuo-spatial mechanisms, we induced changes in self-identification in an IVS phase in which the reflection of the two participants’ faces were merged in the semi-transparent double mirror. Participants performed egocentered and heterocentered visuo-spatial tasks in the pre-stimulation test and repeated these tasks in the post-stimulation test, i.e., after exposure to IVS.

##### Phase 1: Pre-stimulation test

In the pre-stimulation test, one participant was tested in the Self-condition whilst the second participant was simultaneously tested in the Other-condition (see The “Double Mirror” paradigm). In the Self-condition, participants performed a self-centered task that combined egocentered visuo-spatial manipulation and mental own-body transformation with embodied self-location (Arzy et al., 2006; **Self-task)**. In the Other-condition (see The “Double Mirror” paradigm), participants performed an other-centered task that combined heterocentered visuo-spatial manipulation and mental own-body transformation with disembodied self-location (Blanke et al., 2005; Arzy et al., 2006) [**Other-task**], and *vice-versa* depending on blocks.

Only the two green markers lights on the face of the participant tested in the Self-condition were on and emitted light randomly whereas those on the participant tested in the Other-condition were off.

**Self-task (Self-condition**) – In the Self-task, we asked participants to assess whether the green flash that was emitted by the markers on their temples was appearing on the left or right side of their face as reflected in the mirror. The exact instruction was: “You will see your face reflected in the mirror. The markers on your right and left temples will emit green light randomly. Your task consists in assessing whether the green flash is appearing on the right or left side of your face.” Participants were instructed to perform the task while imagining their body at its actual physical position (i.e., using embodied self-location) and keeping their own (egocentered) visuo-spatial perspective, as they are used to when looking at themselves in the mirror.

**Other-task (Other-condition)** – In the Other-task, participants were asked to judge whether the green flash that was emitted by the markers on the temples of their facing partner was appearing on the left or right side of his/her face. The exact instruction was: “You will see the face of your facing partner through the mirror. The markers on his/her right and left temples will emit green light randomly. Your task consists in judging whether the green flash is appearing on the right or left side of your partner’s face.” We instructed participants to perform the tasks while imagining their own body in the body position of their facing partner (i.e., using disembodied self-location) and adopting his/her visuo-spatial perspective (heterocentered).

Green flashes appeared either on the right or left temple for 500 ms. A trial contained one flash and was initiated after a variable interstimulus interval (ISI), randomly chosen between 2000 and 3000 ms (mean 2500 ms). Participants gave their response with a button press on the response box and using their right index finger (**Figure 2B**). Participants could give their response from the stimulus onset and until 1500 ms after the stimulus offset (i.e., 500 ms [stimulus duration] + 1500 ms). Beyond this time limit, a “no-response” was recorded. There were four blocks. Each participant performed the Self- and Other-tasks in two blocks, respectively, and blocks were counterbalanced. Within a block, in a random order, each stimulus (right or left flash) appeared 20 times, giving rise to 40 trials and to a total amount of 160 trials. Each participant performed 80 trials in the Self- and Other-task, respectively.

##### Phase 2: Interpersonal visual stimulation

After completing the baseline visuo-spatial tasks, participants were exposed to the IVS. Before the recording started, we determined for each pair of participants at which frequency the self–other face merging illusory effect was mutually considered as maximal by both participants. This was done while both LEDS sets were alternatively flickering and by modulating the flicker frequencies in a range from 1 to 20 Hz. On average, participants reported that this effect was maximal at 8 ± 1.1 Hz (mean ± SD; 6.4–9.8 Hz; **Table 1**).

**Table 1:** Flicker Frequencies: Interpersonal Visual Stimulation phase.

Pairs of participants	Self–Other merging illusory effect (Hz)
P1-P2	8.6
P3-P4	8
P5-P6	7
P7-P8	9.6
P9-P10	7.8
P11-P12	9.8
P13-P14	7.6
P15-P16	6.4
P17-P18	7.6
**Mean**	**8.04**
***SE***	**1.1**


***Table 1** indicates, for each pair of participants, the flicker frequency that was mutually assessed by both participants as generating the most robust self–other face merging illusory effect. The flicker frequency was determined in the handling procedures before the recording of the Interpersonal Visual Stimulation phase started. We here also report the frequency data for the four participants who were finally excluded from the final data analysis.*

In order that the experimental conditions remain as ecologically valid as possible, the head of the participants was not fixed. Moreover, because of anatomical differences between participants, the position of the two faces was only adjusted with respect to the vertical but not horizontal axis. Before the recording session started, participants were, thus, again instructed to position their face so that the reflection of their eyes matches with that of their partner in the mirror and to maintain this position. Furthermore, they were explicitly instructed not to move, i.e., not to perform lateral movements of their trunk or face but also not to perform contractions of their facial muscles.

In the IVS phase, green flashes appeared randomly either on the face of one or the other participant and either on the left or right temple. We instructed participants to judge whether the green flash on the self–other morphed face as reflected in the mirror was physically appearing on the left or right side of their own face or their partner’s face. The exact instruction was: “You will see your face and that of your partner being merged in the mirror. The markers on your right and left temples and those on your partner’s temples will emit green light randomly, i.e., [1] either on your left or right temple or [2] on the left or right temple of your partner. Your task consists in assessing whether the green flash is appearing on the left or right side of your own face or your partner’s face.”

Participants gave their response with a button press on the serial box. As in the pre-stimulation test, green flashes appeared randomly for 500 ms (ISI: 2000–3000 ms; mean 2500 ms) and participants could give their response from the stimulus onset and until 1500 ms after the stimulus offset. There were four blocks. Within a block, in a random order, each stimulus (right or left flash) appeared 10 times on each participant’s face, giving rise to 40 trials per block and a total amount of 160 trials. In the aggregate, there were 80 Self-trials (i.e., trials in which the green flash physically appeared on one’s own face) and 80 Other-trials (i.e., trials in which the green flash physically appeared on the partner’s face), respectively.

##### Phase 3: Post-stimulation test

Participants in the post-stimulation test performed the same visuo-spatial and mental own-body transformation tasks in the same Self- and Other-conditions as they performed in the pre-stimulation test, i.e., before exposure to IVS (see Phase 1: Pre-stimulation test).

After the recording session, participants were debriefed in order to collect their impression during the IVS-phase. Especially, participants were asked whether they experienced the striking feeling of making one with their facing partner.

### Data Acquisition and Analysis

For each trial in each experimental phase, E-Prime recorded the performance (left/right judgments as indicated by button presses) and reaction times (RTs).

#### Pre-stimulation Test

In this test, we computed for each participant in each Self- and Other-task the total amount and corresponding percentage of correct responses and associated RTs. Because data on performance and RTs were not normally distributed, we calculated Wilcoxon signed-rank tests to test for statistical difference between the Self- and Other-tasks on these two parameters. Concerning performance, statistical measures were done on the number of correct responses.

#### Interpersonal Visual Stimulation

In this phase, we calculated the number and corresponding percentage of errors and absence of response (“no-response”; see Phase 1: Pre-stimulation test and Phase 2: Interpersonal visual stimulation). This was done for the Self- and Other-trials, separately, and then for all the trials pooled together.

We hypothesized that a dominant pattern of attribution errors in reflection symmetry indicates that the self–other face merging illusion is effective. Furthermore, we hypothesized that the egocentered vs. heterocentered visuo-spatial mechanisms in the post-stimulation test would be differently impacted (i.e., impaired vs. improved), depending on the prevailing pattern of attribution errors during exposure to IVS (i.e., attribution errors in reflection symmetry for Self- vs. Other-trials; attribution errors in rotation symmetry for Self- vs. Other-trials). With this aim, the number of errors and corresponding percentage were calculated for each error pattern (see below). We further computed the associated mean RTs for correct responses and each error pattern.

##### Error patterns

There were six potential error patterns in the IVS phase.

**Attribution errors** consisted for participants in either over-attributing the facial features of their own face to their partner’s face (Self → Other) or over-attributing the features of their partner’s face to their own face (Other → Self). That is, for Self-trials, it consisted in assessing that the green flash appeared on the partner’s face whereas it physically appeared on one’s own face. For Other-trials, it consisted in assessing that the green flash appeared on one’s own face whereas it physically appeared on the partner’s face. These attribution errors further distinguish into attribution errors in reflection symmetry (Thirioux et al., 2009; i.e., in mirror reversal; Brugger, 2002) and rotation symmetry (Thirioux et al., 2009; i.e., with preservation of the lateral asymmetry; Brugger, 2002).

**FIGURE 3:**
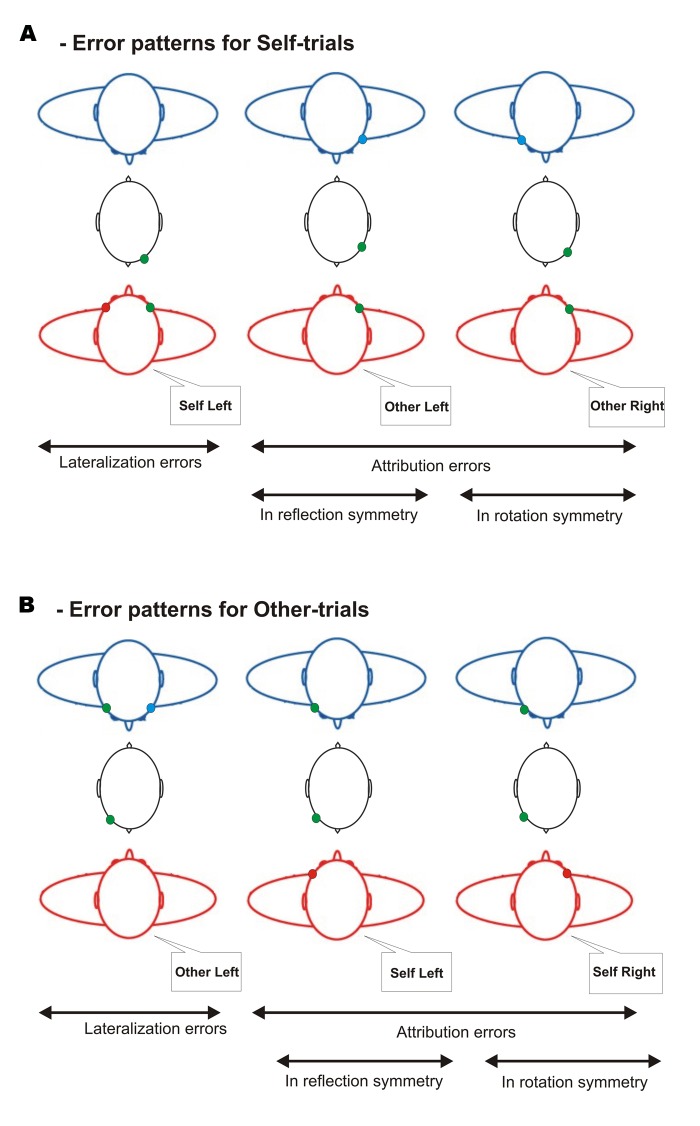
**Error patterns in the Interpersonal Visual Stimulation phase.** **(A)** Error patterns for Self-trials. Lateralization errors consisted in assessing that the green flash appeared on the *left* side of one’s own face whereas it physically appeared on the *right* side. Attribution errors in reflection and rotation symmetry, respectively, consisted in assessing that the green flash appeared on the *left* and *right* side of the partner’s face whereas it physically appeared on the *right* side of one’s own face. And *vice-versa* for flashes appearing on the *left* side of one’s own face. **(B)** Error patterns for Other-trials. Lateralization errors consisted in assessing that the green flash appeared on *the left* side of the partner’s face whereas it appears on the *right* side. Attribution errors in reflection and rotation *symmetry*, respectively, consisted in assessing that the green flash appeared on the *left* and *right* side of one’s own face whereas it physically appeared on the *right* side of the partner’s face. And *vice-versa* for flashes appearing on the *left* side of the partner’s face.

Attribution errors in **reflection symmetry** (A-Ref errors) consisted for **Self-trials** in assessing that the green flash appeared on the *left* side of the partner’s face whereas it physically appeared on the *right* side of one’s own face, and *vice-versa* (right–left; **Figure 3A**). For **Other-trials**, it consisted in assessing that the green flash appeared on the *left* side of one’s own face whereas it physically appeared on the *right* side of the partner’s face, and *vice-versa* (right–left; **Figure 3B**).

**FIGURE 4:**
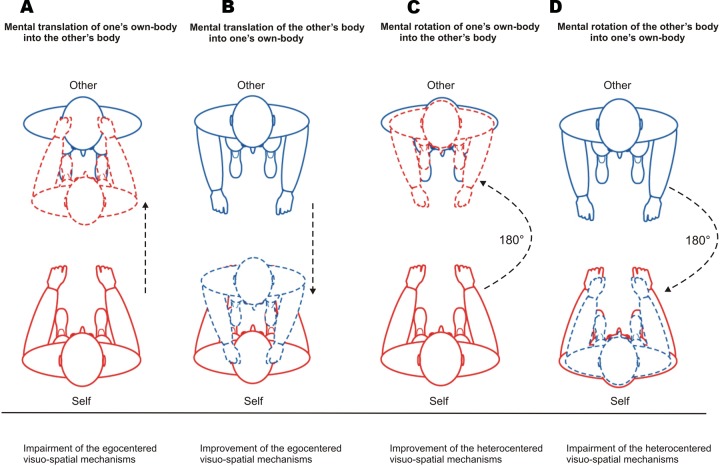
**Patterns of attribution errors with respect to visuo-spatial mechanisms and mental body transformations.** **(A)** Attribution errors in reflection symmetry for Self-trials indicate that participants have identified their partner’s face with their own face by mentally translating their own-body in a linear fashion toward the other’s body. **(B)** Attribution errors in reflection symmetry for Other-trials indicate that participants identified their face with their partner’s face by mentally translating the other’s body in a linear fashion toward their own-body but without matching between the bodies’ axes. **(C)** For Self-trials, attribution errors in rotation symmetry reflect that participants adopted the visuo-spatial perspective of their partner (heterocentered mechanism) and felt themselves as being located in their facing partner’s body position (disembodied self-location). That is, they incorporated the other’s facial features into their own face by mentally rotating their own-body by 180° into their partner’s body and aligning their own-body axis on the body axis of their partner. **(D)** Attribution errors in rotation symmetry for Other-trials indicate that participants have incorporated the other’s facial features into the representation of their own face by mentally rotating the other’s body by 180° into their own-body and aligning the other’s body axis on their own body axis.

We hypothesized that attribution errors in reflection symmetry for Self-trials (Self → Other) impairs the egocentered visuo-spatial mechanisms. These errors would reflect that participants have identified their partner’s face with their own face as if their own face were the reflection of the other’s face. That is, they would have projected their own facial features toward their partner’s face by mentally translating their own-body in a linear fashion toward the other’s body. This pattern of errors, although it does not involve heterocentered perspective change, requires that participants feel themselves toward the other, i.e., do not feel themselves in their actual embodied self-location and egocentered reference frame. Accordingly, egocentered visuo-spatial mechanisms would be impaired (**Figure 4A**).

In contrast, we hypothesized that attribution errors in reflection symmetry for Other-trials (Other → Self) improve the egocentered visuo-spatial mechanisms. These errors would reflect that participants have identified their face with their partner’s face as if the other’s face were the reflection of their own face. That is, they would have introjected the facial features of their partner in their own face by mentally translating his/her body in a linear fashion toward their own-body but without matching between the bodies’ axes. Accordingly, this pattern of errors occurring in an embodied self-location and egocentered visuo-spatial perspective would reinforce these mechanisms (**Figure 4B**).

Attribution errors in **rotation symmetry** (A-Rot errors) consisted for **Self-trials** in assessing that the green flash appeared on the *right* side of the partner’s face whereas it physically appeared on the *right* side of one’s own face, and *vice-versa* (left–left; **Figure 3A**). For **Other-trials**, it consisted in assessing that the green flash appeared on the *right* side of one’s own face whereas it physically appeared on the *right* side of the partner’s face, and *vice-versa* (left–left; **Figure 3B**).

We hypothesized that attribution errors in rotation symmetry for Self-trials improve the heterocentered visuo-spatial mechanisms. These errors would reflect that participants adopted the visuo-spatial perspective of their partner (heterocentered mechanism) and felt themselves as being located in their facing partner’s body position (disembodied self-location). That is, they would have incorporated the other’s facial features into their own face by mentally rotating their own-body by 180° into their partner’s body and aligning their own-body axis on the body axis of their partner (**Figure 4C**).

In contrast, we hypothesized that attribution errors in rotation symmetry for Other-trials impaired the heterocentered coding because they rely upon egocentered visuo-spatial mechanisms and self-location processes. In fact, these errors would reflect that participants have incorporated the other’s facial features into the representation of their own face by mentally rotating the other’s body by 180° into their own-body and aligning the other’s body axis on their own body axis (**Figure 4D**).

**Lateralization errors** consisted for **Self-trials** in assessing that the green flash appeared on the *left* side of one’s own face whereas it physically appeared on the *right* side and *vice-versa* (right–left; **Figure 3A**). For **Other-trials** it consisted in assessing that the green flash appeared on *the left* side of the partner’s face whereas it appears on the *right* side, and *vice-versa* (right–left; **Figure 3B**).

To test which errors pattern prevailed during exposure to IVS, we computed a Kruskal–Wallis test on the number of errors between error patterns. To further test whether the number of correct responses and errors for each error pattern differed between Self- and Other-trials, we calculated Wilcoxon signed-rank tests. Finally, to test for RTs differences between conditions (Self- vs. Other-trials), we calculated Wilcoxon signed-rank tests. This was done for the correct responses and each pattern of errors.

#### Post-stimulation Test

In this test, we computed the total amount and corresponding percentage of correct responses, and associated RTs for each participant in each task, as in the pre-stimulation test. To test for statistical analyses between the Self- and Other-task, we calculated Wilcoxon signed-rank tests on performance (number of correct response) and RTs.

Then, to statistically test whether the IVS-phase impacted the egocentered or heterocentered visuo-spatial mechanisms, we computed a 2 × 2 repeated-measures ANOVA with permutation tests on the number of correct response and RTs with time (pre-IVS vs. post-IVS) and tasks (Self-task vs. Other-task) as the factors. We calculated the statistical power which was about 0.71 (1 – β).

As mentioned above (see Participants), we excluded the data from four participants [participants 9 (P9), 10 (P10), 14 (P14), and 17 (P17)] because of bad recording conditions in the IVS-phase. During the debriefing (see Phase 3: Post-stimulation test), P9 and P10 (tested in the same recording session) reported that they intentionally performed slight lateral head movements in the IVS-phase. P14 and P17 (tested in different recording sessions) reported that they performed slight contradictions of facial muscles. According to these participants, these strategies helped them to discriminate more easily between their own face and that of their partner and the self–other merging illusion was not effective. Corroborating their self-report, the data analysis indicated that these participants obtained in average 72.5 ± 8% (mean ± SE) of correct responses in the IVS-phase although the mean percentage of correct responses was about 27.3 ± 3% in the remaining fourteen participants. Consequently, the final analyses on the pre-stimulation, IVS and post-stimulation tests were only performed on the data from the remaining fourteen participants (**Table 2**).

**Table 2:** Interpersonal Visual Stimulation phase: correct performance.

Participants	Correct Performance %
P1	22.5
P2	40.6
P3	20
P4	23.1
P5	48.1
P6	23.8
P7	36.9
P8	43.1
P11	36.9
P12	23.1
P13	16.3
P15	31.2
P16	16.3
P18	0.6


***Table 2** reports the percentage of correct responses during exposure to IVS for the remaining 14 participants.*

## Results

### Pre-stimulation Test (Phase 1)

In the pre-stimulation test, participants performed correctly for the Self- and Other-tasks, using egocentered visuo-spatial mechanisms in the Self-task (98.6 ± 0.5%; mean ± SEM) and heterocentered visuo-spatial mechanisms in the Other-task (97.3 ± 0.1%), respectively. Correct performance did not differ between tasks (*p* = 0.300).

Concerning response speed, participants performed significantly faster in the Self- (541 ± 30 ms) than Other-task (686 ± 41 ms) (*p* < 0.001).

### Interpersonal Visual Stimulation (Phase 2)

**FIGURE 5:**
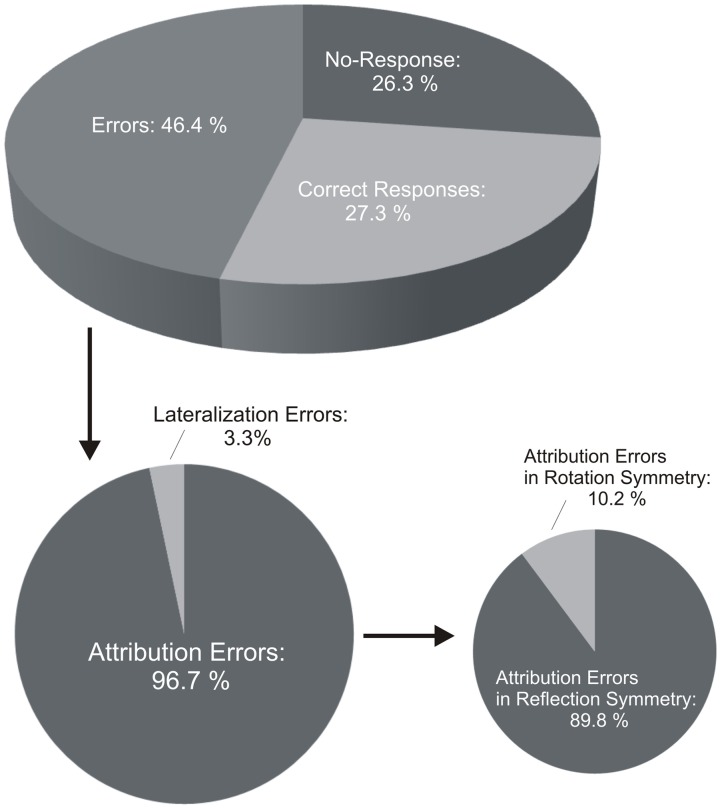
**Percentages of correct performance and errors in the Interpersonal Visual Stimulation phase.** Participants in the IVS phase performed correctly in ∼27% of the trials. There were ∼46% of errors. Attribution errors highly prevailed in comparison to lateralization errors. Percentage of attribution errors in reflection symmetry, i.e., in mirror like-fashion, was significantly higher than in rotation symmetry, i.e., with preservation of the lateral asymmetry.

On average, participants performed correctly in 27.3 (±3.4) % of the trials (pooled data from Self- and Other-trials). There was 26.3 (±3.6) % of no-response and 46.4 (±4.2) % of errors (**Figure 5**). There was no statistical difference between Self- and Other-trials (all *p* > 0.05).

On the total amount of errors, participants performed more attribution (96.7 ± 3.8%) than lateralization errors (3.3 ± 0.4%) (**Figure 5**). Regarding attribution errors, there were more errors in reflection (89.8 ± 3.6%) than rotation symmetry (10.2 ± 1.5%). This was statistically confirmed by a Kruskal–Wallis test (*p* < 0.001) and paired comparisons [A-Ref errors vs. A-Rot errors (*p* < 0.001); A-Ref errors vs. Lat-errors (*p* = 0.076)]. This was true for both Self-trials [Kruskal–Wallis, *p* < 0.001; paired comparisons, A-Ref errors vs. A-Rot errors (*p* = 0.001), A-Ref errors vs. Lat-errors (*p* < 0.001), A-Rot errors vs. Lat-errors (*p* = 0.446)] and Other-trials [Kruskal–Wallis, *p* < 0.001; paired comparisons, A-Ref errors vs. A-Rot errors (*p* < 0.001), A-Ref errors vs. Lat-errors (*p* < 0.001), I-Rot errors vs. Lat-errors (*p* = 0.819)], when considered separately (**Figure 5**). Wilcoxon signed-rank tests further showed that the number of errors with respect to each error pattern did not statistically differ between Self- and Other-trials (A-Ref errors, *p* = 0.401; A-Rot errors, *p* = 0.910; Lat-errors, *p* = 0.511; **Figure 5**).

Concerning RTs, there was no statistical difference between Self- and Other-trials for correct responses (*p* = 0.650). This was also true for each pattern of errors (all *p* > 0.050).

### Post-stimulation Test (Phase 3)

In the post-stimulation test, participants performed correctly for the Self-task (95.6 ± 1%) and Other-task (95.6 ± 1.2%). RTs were faster in the Self- (576 ± 35 ms) than Other-task (680 ± 35 ms) (*p* < 0.001).

**FIGURE 6:**
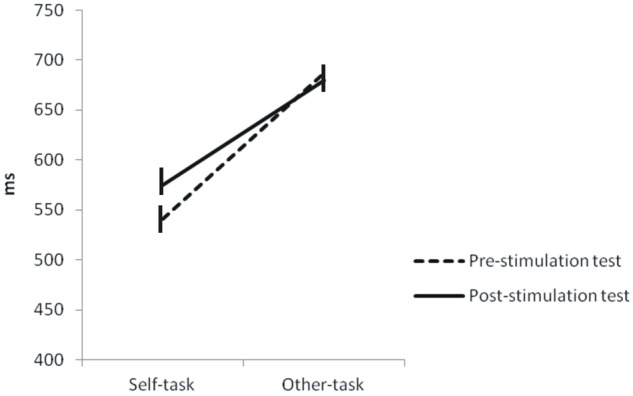
**Comparison of reaction times between the pre- and post-stimulation tests.** Statistical comparisons showed that RTs significantly increased in the post-stimulation test in comparison to the pre-stimulation test. This was also observed for the Self-task but not for the Other-task.

Statistical comparisons on RTs showed a significant time × task interaction (*p* = 0.0444) and a significant effect of tasks (*p* < 0.001). There was no effect of time (*p* = 0.389). These indicate that RTs significantly increased in the post-stimulation test (576 ± 35 ms) in comparison to the pre-stimulation test (541 ± 30 ms) selectively for the Self-task (**Figure 6**). Concerning performance, there was a significant effect of time (*p* = 0.044) but no time × task interaction and no effect of task.

## Discussion

In the present study, we investigated whether identifying oneself with the face of another individual in the mirror impacts either the egocentered or heterocentered visuo-spatial mechanisms that sustain self–other distinction. For that, self-identification was manipulated by exposing participants to an IVS condition in which the reflection of their own face was merged with that of their facing partner in a semi-transparent double mirror. In the pre- and post-IVS tests, participants performed egocentered visuo-spatial transformations with respect to their own face as reflected in the mirror and heterocentered visuo-spatial transformations with respect to their partner’s face seen through the mirror. Our data show that self–other face identification alters the egocentered visuo-spatial mechanisms but preserved the heterocentered coding. This suggests that changes in self-face identification induced a conflict between the current visual representation and stored mnemonic representation of the self-face which, in turn, impacted bodily self-consciousness. We discussed the interaction of bottom-up and top-down processing factors in self-face identification on the basis of the nested hierarchy model of the Self (Feinberg, 2000).

### Self-face Prioritization Effect in Egocentered Visuo-spatial Manipulation Task

In the pre-stimulation test, participants performed correctly for the egocentered (Self-condition) and heterocentered (Other-condition) visuo-spatial tasks. Mean percentages of correct responses did not differ between tasks (Self-task: ∼98.6%; Other-task: ∼97.3%). In contrast, RTs were significantly faster for the Self-task (∼541 ms) than Other-task (∼686 ms).

Firstly, these faster RTs in the Self-task are possibly explained by the SFP effect. This prioritized processing of one’s own face is generated by visual self-recognition processes based upon view-invariant representations of the face (Tsakiris, 2008) and by mnemonic representations, attentional processes, retrieval of semantic and/or autobiographical memories and higher-order self-evaluation processes (Sui et al., 2015). On a neuro-functional level, SFP is reflected during self–other face discrimination tasks in greater activations in a dedicated bilateral fronto-parietal network, including the inferior parietal lobule (IPL), inferior, middle and medial frontal cortices, IOG, and temporal gyrus (Caharel et al., 2002; Uddin et al., 2005a,b; Northoff et al., 2006; Platek et al., 2006, 2008; Devue and Brédart, 2011; Sui et al., 2015) as well as in an increased amplitude of the face-sensitive N170 occipito-temporal component (Keyes et al., 2010). However, contrasting with studies on SFP, we here did not use Self- vs. Other-face discrimination tasks in the pre-stimulation phase. This suggests that this potential SFP effect in our data was rather due to mere implicit recognition processes, i.e., that do not require to explicitly discriminate between Self and Others.

Secondly, this difference in response speed is also typical for egocentered vs. heterocentered visuo-spatial tasks and conforms to previous behavioral studies employing comparable mental own-body transformation tasks (Parsons, 1987; Zacks et al., 1999; Blanke et al., 2005; Arzy et al., 2006; Mohr et al., 2006; Easton et al., 2009; Thirioux et al., 2009, 2010, 2014b). Computing heterocentered visuo-spatial judgments triggers an increase of RTs in comparison to egocentered visuo-spatial computation. In fact, the former necessitate to mentally aligning one’s own-body axis on the other’s body axis (Arzy et al., 2006). This effect is further greater when the position of the other’s body in space does not match that of one’s own body, as in face-to-face postural configurations (Thirioux et al., 2009, 2010, 2014b). Accordingly, imagining oneself to be located in the body position of a front-facing individual, as it was requested in the Other-task, requires a mental rotation by 180° of one’s own-body. This specific visuo-spatial and mental own-body transformation is cognitively more demanding than computing visuo-spatial judgments centered on one’s own-body as the latter do not need mental own-body or egocentered transformation.

Therefore, a combination of visual-mnemonic processing of the face and visuo-spatial processing of the body may account for our RTs data. We suggest that both intentional object – i.e., one’s own face vs. the other’s face – and nature of the requested cognitive tasks – i.e., visuo-spatial judgments centered on one’s own vs. other’s body – may explain theses faster RTs in the Self- than Other-task. This hypothesis is reinforced by neuroimaging data, showing that face- and body-recognition triggers co-activations in the right insular and parietal cortices (Kircher et al., 2001; Ehrsson et al., 2004; Devue et al., 2007; Tsakiris et al., 2007). These co-activations in brain regions that underpin multisensory processing, specifically in the right hemisphere, suggest that face- and body-recognition share more general self-related processes (Tsakiris, 2008). Therefore, our findings tend to reflect the phenomenological status of the self as first and immediate reference frame, speaking in favor of an implicit self-advantage theory (Ma and Han, 2010; Ferri et al., 2011; Geng et al., 2012; Tacikowski and Ehrsson, 2016).

### Spontaneous Mirror-Like Self–Other Identification during Exposure to Interpersonal Visual Stimulation

Exposure to the IVS followed the completion of the baseline visuo-spatial tasks. During IVS, participants saw the reflection of their own face that was merged with that of their facing partner in the mirror. They were instructed to assess whether the green flash on the self–other morphed face as reflected in the mirror was physically appearing on the left or right side of their own face or that of their partner. Participants were significantly impaired in responding to the requested tasks and performed correctly in about 27% of the trials. The errors rate reached about 46% and the no-response rate about 26%. Attribution errors highly prevailed (∼97%), in comparison to lateralization errors (∼3%). Furthermore, there were significantly more attribution errors in reflection (∼90%) than rotation symmetry (∼10%), i.e., in mirror reversal than with preservation of the lateral asymmetry. The mean amount of attribution errors in both reflection and rotation symmetry did not statistically differ between Self-trials (i.e., when the green flash physically appeared on one’s own face) and Other-trials (i.e., when the green flash physically appeared on the other’s face).

Firstly, this dominant pattern of attribution errors, compared to lateralization errors, shows that participants had more difficulties in correctly recognizing which face (i.e., their own or that of their partner) was physically targeted by the green flash than in correctly locating the green flash *per se* (right vs. left side of the face). Moreover, the prevailing pattern of attribution errors in reflection symmetry in particular conforms to our working hypothesis that self–other identification is associated with mirror-like behaviors.

Secondly, this high prevalence of mirror-like behaviors is an interesting finding because this behavioral pattern arised spontaneously between participants. In fact, self-identification with the other’s face in previous studies is traditionally elicited by the use of IMS that is intentionally designed in a mirror-like fashion (e.g., Tsakiris, 2008; Sforza et al., 2009). Such design aims to elicit a specular correspondence between the touch that is felt by the participant on his/her cheek and the synchronous “touching” paintbrush that is seen on the other’s cheek. As an example, the tested participant is feeling a touch on his/her right cheek while seeing a paintbrush touching synchronously the left cheek of the other’s face (Sforza et al., 2009). In our experiment, and although we did not use interpersonal multisensory – i.e., visuo-tactile – but unisensory – i.e., visual – stimulation, this mirror-like behavior occurred spontaneously between participants. That is, although we did not bias self–other identification face toward this specular modality.

Thirdly, our data show that attribution errors in reflection symmetry did not differ between Self- and Other-trials. This suggests that participants have over-attributed their own facial features to the other’s face (Self → Other) as well as having over-attributed the other’s facial features to their own face (Other → Self). That is, they have projected the features of their face toward the other’s face as many as having introjected the features of their partner’s face toward their own face. We here propose that this absence of difference between projection- and introjection-based processes indicates a bidirectional self–other face identification. This bidirectional self- and other-facial features attribution in a specular correspondence probably generated a balanced self–other confusion effect. That is, a self–other confusion that was not determined by a unidirectional over-inclusion of self- or other-attributes.

Taken together, our data demonstrate that our newly developed paradigm has many advantages for the investigation of self-consciousness and self–other distinction. It enables generating a robust self–other face merging illusory effect in ecologically more valid conditions, i.e., when two individuals are physically facing each other and interacting. It significantly distinguishes, thus, from paradigms as used in previous studies that are most often based upon pre-recorded static images or movies of progressive morphing from self- to other-face and *vice-versa* (Tsakiris, 2008; Tajadura-Jiménez et al., 2012; Maister et al., 2013; Apps et al., 2015). Moreover, our paradigm enables eliciting self–other identification experience from an interpersonal unisensory (visual) stimulation. Finally our paradigm provides empirical criteria that are based upon objective measures (RTs and percentages of performances) of self–other face identification. That is, measures that are not only based upon self-rated (subjective) judgments as usually employed (Sforza et al., 2009; Maister et al., 2013).

### Self-face Identification Contributes to Bodily Self-consciousness

In the post-stimulation test, i.e., after exposure to IVS, participants performed the same Self-task (egocentered visuo-spatial mechanisms) and Other-task (heterocentered visuo-spatial mechanisms) as in the pre-stimulation test. Comparable to pre-IVS, participants performed significantly faster in the Self- (∼576 ms) than Other-task (∼680 ms). Interestingly enough, visual comparison of the pre- and post-IVS data suggested that RTs increased after IVS but selectively for the Self-task. This effect was confirmed by statistical comparisons, showing a significant time (pre-IVS vs. post-IVS) × task (Self-task vs. Other-task) interaction that was attributable to a significant difference between the pre-IVS Self-task and the post-IVS Self-task. RTs for the Other-task did not differ after exposure to IVS in comparison to the pre-stimulation phase. Therefore, our data indicate that participants were slow-downed and had more difficulties in performing own-body centered and egocentered visuo-spatial judgments after than before exposure to the stimulation. This suggests that the self–other identification during IVS specifically impaired the egocentered visuo-spatial mechanisms but preserved the heterocentered coding.

It could be objected that the prioritized processing of the self-face was lost in the post-stimulation phase because participants performed the task twice. Accordingly, the observed effect on self-related RTs would not have been caused by the exposure to IVS but by the repetition of the task, i.e., by long and more difficult perceptual conditions. This would have triggered an increase in response time and self-related stimuli would have been more impacted than other-related stimuli. This would be also concordant with the significant effect of time on performance, showing that participants performed more errors for both Self- and Other-tasks in the post- than pre-stimulation phase. However, this objection is not tenable. Firstly, if so, other-related stimuli RTs should have also been affected by the repetition of the task and prolonged exposure to the same task. Secondly, because heterocentered visuo-spatial computation is cognitively more demanding than egocentered visuo-spatial computation, other-related stimuli should therefore have more suffered from the repetition of the task than self-related stimuli. Which was not the case: RTs in the Other-task did not differ between the pre- (∼686 ms) and post-stimulation (∼680 ms).

Previous behavioral findings (Tsakiris, 2008; Sforza et al., 2009; Tajadura-Jiménez et al., 2012; Maister et al., 2013) demonstrated that IMS affected both self-face recognition and representation whereas the recognition and representation of the other’s face remained unchanged (Maister et al., 2013). It is worth noting that our experimental design differs from that of previous studies. We here investigated the effect of a visual conflict between one’s own face and the face of another individual on visuo-spatial mechanisms whilst prior studies investigated the effect of a visuo-tactile conflict between the other’s face and one’s own face on self-face recognition. However, despite these differences, our data showing that egocentered visuo-spatial mechanisms were impaired after exposure to IVS whereas heterocentered visuo-spatial mechanisms were preserved, converge in a general way with these prior results. Considered collectively, data show that a situation of self–other face merging illusion, be it caused by multisensory (visuo-tactile) [previous work] or unisensory (visual) stimulation [present work], alters self-related processing but preserves other-related processing. This is true whether self-related processing concerns face-recognition [previous work] or egocentered visuo-spatial mechanisms [present work].

It should be acknowledged that the difference on RTs (+35 ms) between the pre- and post-stimulation tests in our data is tenuous. However, prior studies, even though using different behavioral parameters, also reported low differences between tasks that were performed before and after stimulation. For instance, studies by Tsakiris (2008) and Tajadura-Jiménez et al. (2012) have, respectively, shown that images of self–other morphed face that contained an average 5.6% and 3% more of facial other-features were assessed as self-face in the post-stimulation test, compared to the pre-stimulation test. These tenuous differences in our data and these of other groups, although statistically significant, may reflect the robustness of the neural self-representation and solidity of the self as first-reference frame in normal subjects. On a more general level, our data indicate that a transitory visual self–other confusion impairs, although slightly, the mechanisms of egocentered spatial coding that underpins both self-location and first-person perspective.

Initially, we hypothesized that a prevailing pattern of attribution errors in reflection symmetry for Self-trials vs. Other-trials, respectively, impairs and improves the egocentered visuo-spatial mechanisms. Our data are only partly in accordance with this working hypothesis. Firstly, as mentioned earlier, participants identified in a bidirectional manner their own face with that of their partner and the face of their partner with their own face. That is, participants have introjected their partner’s facial features toward their own face (Other → Self; Self-trials) as many as having projected their own facial features toward their partner’s face (Self → Other; Other-trials). Secondly, we observed an increase of RTs that was selective for the Self-task after exposure to IVS. Considered collectively, these results suggest that attribution errors in reflection symmetry for Self-trials impaired the egocentered visuo-spatial mechanisms, verifying our hypothesis. However, these further suggest that attribution errors in reflection symmetry for Other-trials not only did not improve but impaired the egocentered coding, contradicting our hypothesis.

This indicates that projection- and introjection-based processes equally altered embodied self-location and egocentered visuo-spatial mechanisms because they equally disturbed the Self boundaries in our study. That is, these two processes equally transformed the margins between the Self and the Other in either externalizing (Freud, 1930) self-face features into the other (projection) or alienating (Freud, 1930) facial features that do not belong to the self but to the other (introjection; Feinberg and Keenan, 2005). These results are in accordance with the Freudian view that “the boundaries of the ego are not constant” (Freud, 1930). Therefore, our results confirm that body-related information needs to be continually updated for the maintenance of the sense of the Self in line with previous studies. But they also show that self-face identification plays a major role in this continual body-related information updating. Accordingly, this association between a bidirectional self–other identification that was based upon projection and introjection processes in the IVS-phase and impairment of the egocentered visuo-spatial mechanisms in the post-IVS test indicates that self-face identification importantly contributes to bodily self-consciousness. To the best of our knowledge, we raised here a novel phenomenological and functional aspect of the relation between self-face identification and bodily self-consciousness insofar as previous studies have mainly shown, inversely, how bodily self-consciousness contributes to self-face identification.

It is well-documented that recognizing one’s own face in the mirror is an important behavioral marker of higher-order consciousness (Apps et al., 2015; see also Keenan et al., 2005). Self-face recognition firstly requires that the current perception of the seen face’s surface structure that is based upon visual-attentional processing matches with the kinesthesic representation of one’s own-body. Secondly, it also necessitates that this multisensory integration-based representation matches with a stored representation of one’s own face in the perceptual memory system (Devue and Brédart, 2011). Higher-order self-related semantic and autonoetic representations may be then retrieved, enabling to access an amodal representation of the self and validating, in turn, the belief that the reflection seen in the mirror “is me” (Sugiura et al., 2014; see also Sugiura et al., 2012). We here further suggest that during exposure to IVS the conflict between the current visual representation of one’s own face and the stored representation of the self-face in the perceptual memory system has elicited projection and introjection-based behavioral responses. These behaviors have significantly disturbed the Self boundaries as reflected in alteration of the egocentered visuo-spatial mechanisms in the post-stimulation test. Therefore, it would mean that changes in the current visual representation of one’s own face induced a mismatching with the stored mnemonic representation of the self-face which, in turn, impacted bodily self-consciousness.

According to the nested hierarchy model of the Self (Feinberg, 2000, 2001, 2011; Feinberg and Keenan, 2005), lower-order features are “nested within” higher-order features, i.e., are bound together and dependent from each other with respect to bottom-up and top-down dynamic processing. This enables the emergence of the Self as a more complex whole (Feinberg and Keenan, 2005). According to this model, self-face identification would be based upon an interaction between bottom-up flow of information, from perceptual to higher-order cognitive processes, but also top-down processing. Our data may be in line with this dynamic model in suggesting that self-face mnemonic representation, from the processing of a conflicting bottom-up flow of information, may have top-down impacted lower-order bodily self-consciousness.

To sum up it all, the present study shows for the first time the impact of self-face identification on the egocentered visuo-spatial mechanisms. It further indicates that the mnemonic self-representation importantly contributes to bodily self-consciousness. On a neuro-functional level, we hypothesize that changes in the IOG activity during the IVS phase triggered a modulation of the activation in the right frontal areas (notably inferior frontal gyrus and medial frontal cortex) that, in turn, top-down modulated the right TPJ activity (supporting egocentered and first-person perspective). Moreover, we hypothesize that this activation modulation in the frontal cortex preserved the left TPJ activity (supporting heterocentered and second-person perspective). It would be of great interest to test these neuro-functional hypotheses in future experiments, using the same paradigm and during EEG hyperscanning recording. Extending previous findings (Tsakiris, 2008; Sforza et al., 2009; Apps et al., 2012, 2015; Tajadura-Jiménez et al., 2012; Maister et al., 2013; Preston et al., 2015), our present results suggests that not only bodily self-consciousness contributes to self-face identification but that self-face identification also plays a major role in bodily self-consciousness.

Concerning bodily self-consciousness, our data also highlight the importance of the egocentered visuo-spatial processing in the distinction between self and others. This is further concordant with the phenomenological hypothesis that bodily self-consciousness, under non-pathological conditions, lies at the basis of a balanced self–other relationship (Langdon and Coltheart, 2001; Thirioux, 2011). This also corroborates the view that bodily self-consciousness dysfunctions, i.e., a poor somaesthetic insight (Jaafari and Markova, 2011), contribute to generate disorders of social relationships (Langdon et al., 2001) and empathic process in psychiatric diseases such as schizophrenia (Thirioux and Jaafari, 2015). Such disturbances in empathic and self–other distinction processes are reflected in either difficulty in disengaging from oneself as first-reference frame or in over-exaggerated facility in inhibiting one’s egocentered perspective in patients with, respectively, prevailing negative and positive symptoms (Thirioux et al., 2014b).

## Conclusion

In the present study, we report that self-identification with the face of another individual alters the egocentered visuo-spatial mechanisms but preserves the heterocentered coding. In accordance with an implicit self-advantage theory, our findings suggest that self-identification contributes to bodily self-consciousness. These further reinforce new theoretical views and experimental data according to which self-face identification and bodily self-consciousness share perceptual-cognitive and neurobiological units. Our data seem to confirm the originality as well as solidity of our newly developed experimental paradigm. Moreover, our paradigm may be used to investigate disorders of self-consciousness, sense of identity and self–other distinction in psychiatric and neurological diseases and also in remediation protocols that aim to improve such deficits.

## Author Contributions

MW designed the mirror as an installation and programmed the Double Mirror System. AB initiated the use of the Double Mirror for scientific testing. BT and AB conceived the paradigm of visuo-spatial reference frame manipulation and designed the experiment. BT and MW conducted the experiment. BT analyzed the data. BT, NJ, and AB discussed and interpreted the results. NL programmed the repeated measures ANOVA with permutation tests. BT and AB wrote the manuscript.

## Conflict of Interest Statement

The authors declare that the research was conducted in the absence of any commercial or financial relationships that could be construed as a potential conflict of interest.
